# *Xist* reduction in breast cancer upregulates AKT phosphorylation via HDAC3-mediated repression of PHLPP1 expression

**DOI:** 10.18632/oncotarget.9673

**Published:** 2016-05-27

**Authors:** Yen-Sung Huang, Che-Chang Chang, Szu-Shuo Lee, Yuh-Shan Jou, Hsiu-Ming Shih

**Affiliations:** ^1^ Institute of Biomedical Sciences, Academia Sinica, Taipei, Taiwan; ^2^ Graduate Institute of Translational Medicine, College of Medical Science and Technology, Taipei Medical University, Taipei, Taiwan; ^3^ Program in Molecular Medicine, National Yang-Ming University and Academia Sinica, Taipei, Taiwan

**Keywords:** lncRNA, Xist, AKT, HDAC3, breast cancer

## Abstract

Long noncoding RNAs (lncRNAs) dysregulated in cancer potentially play oncogenic or tumor-suppressive roles. While the X inactivate-specific transcript (*Xist*) lncRNA is important for X-chromosome inactivation in female cells, very little is known about the role of *Xist* in human breast cancer in modulating cellular pathway(s). Here, we show that *Xist* expression is significantly reduced in breast tumor samples and cancer cell lines. *Xist* knockdown or overexpression resulted in increased or decreased levels, respectively, of AKT phosphorylation and cell viability. Further studies revealed an inverse correlation between *Xist* and phospho-AKT levels in breast cancer samples. Additionally, *Xist* knockdown-elicited increase of cell viability was attenuated by AKT inhibitor. These results suggest that *Xist* negatively regulates cell viability via inhibition of AKT activation. Interestingly, decreased *Xist* expression in breast cancer samples was associated with reduced levels of *Jpx* RNA, an lncRNA that positively regulates *Xist* promoter activity. Accordingly, *Jpx* knockdown enhanced AKT activation and cell viability. We also demonstrate that knockdown of *Xist* or SPEN, an intermediator protein to link *Xist*, SMRT co-repressor and HDAC3 complexes for X-chromosome inactivation, decreased expression of PHLPP1, a phosphatase to remove AKT phosphorylation, via increased HDAC3 recruitment to the PHLPP1 promoter, correlating with increased AKT phosphorylation. Our findings elucidate the tumor suppressor role of *Xist* in breast cancer and provide the molecular basis of *Xist* in downregulating AKT activation.

## INTRODUCTION

Long noncoding RNAs (lncRNA) are non-protein coding transcripts of more than 200 nucleotides [1]. In recent years, reports of dysregulated lncRNA expression in numerous tumor types suggest that lncRNAs may act as potential oncogenic or tumor-suppressive RNAs [2]. The X inactivate-specific transcript (*Xist)*, a 19 kb lncRNA (17 kb in mouse) from the inactive X-chromosome (Xi), is required for X-chromosome inactivation (XCI) in female cells [3]. However, the role of *Xist* as an oncogenic or a tumor-suppressive lncRNA remains largely unclear [4]. *Xist* loss in hematopoietic stem cells was shown to result in the development of female-specific highly aggressive myelodysplastic syndrome (MDS) and myeloproliferative neoplasm (MPN), suggesting a tumor suppressor role for *Xist* [5]. In contrast, a recent report indicated an oncogenic role of *Xist* in glioblastoma stem cells by evidence that *Xist* expression was elevated in glioma tissues and that *Xist* knockdown reduced cell proliferation, migration and invasion in glioblastoma stem cells [6]. Interestingly, *Xist* expression was reduced in some breast cancer cell lines compared to normal cell lines [7]. Very little is known, however, about whether *Xist* plays an oncogenic or a tumor suppressive role in human breast cancer.

*Xist* expression is positively and negatively regulated by noncoding *Jpx* RNA and antisense *Tsix* RNA, respectively. *Jpx* RNA could bind CTCF protein and titrate out the repression effect of CTCF on *Xist* promoter [8]. Conversely, *Tsix* RNA could facilitate PRDM14 binding to *Xist* intron 1 to suppress its expression [9]. In addition, pluripotency factors such as OCT4, SOX2, REX1 and NANOG could bind to *Xist* intron 1 for transcriptional repression, while these factors also activate *Tsix* expression [10]. Currently, the regulation of decreased *Xist* levels in breast cancer cells has not been fully elucidated.

*Xist* plays a crucial role in XCI processes, initially binding to the inactive X-chromosome *in cis* inducing a cascade of events, including XCI establishment (such as euchromatin mark removal and RNA polymerase II exclusion) and XCI maintenance (such as repressive chromatin modification formation and DNA methylation) [11]. The recruitment of numerous repressive complexes by *Xist*, such as polycomb repressive complex 2 (PRC2) and DNA-methyltransferase, are required for the XCI maintenance [8]. Two research groups have recently implicated *Xist* RNA interactors, including SPEN/SHARP, in XCI establishment [12, 13]. The SPEN/SHARP protein directly interacts with the SMRT co-repressor, leading to the recruitment of HDAC3 and further activation of HDAC3 activity in removing euchromatin marks and excluding RNA polymerase II across the X chromosome [12, 14]. While *Xist* plays an important role in XCI processes, it remains to be elucidated whether decreased *Xist*-regulated expression of genes, other than in the X chromosome, are associated with breast cancer.

Here, we demonstrate *Xist* acts as a tumor-suppressor lncRNA in breast cancer cells by decreasing AKT phosphorylation. Expression of *Jpx* and *Xist* were downregulated in breast tumor. Knockdown of either *Xist* or SPEN expression in breast cancer cells suppressed the expression of PHLPP1, a phosphatase in AKT dephosphorylation [15], and was correlated with increased HDAC3 recruitment to the PHLPP1 promoter. Our findings provide a previously undescribed molecular basis of *Xist* in suppressing the AKT pathway in breast cancer.

## RESULTS

### *Xist* expression is significantly reduced in breast cancer cell lines and breast cancer samples

**Figure 1:**
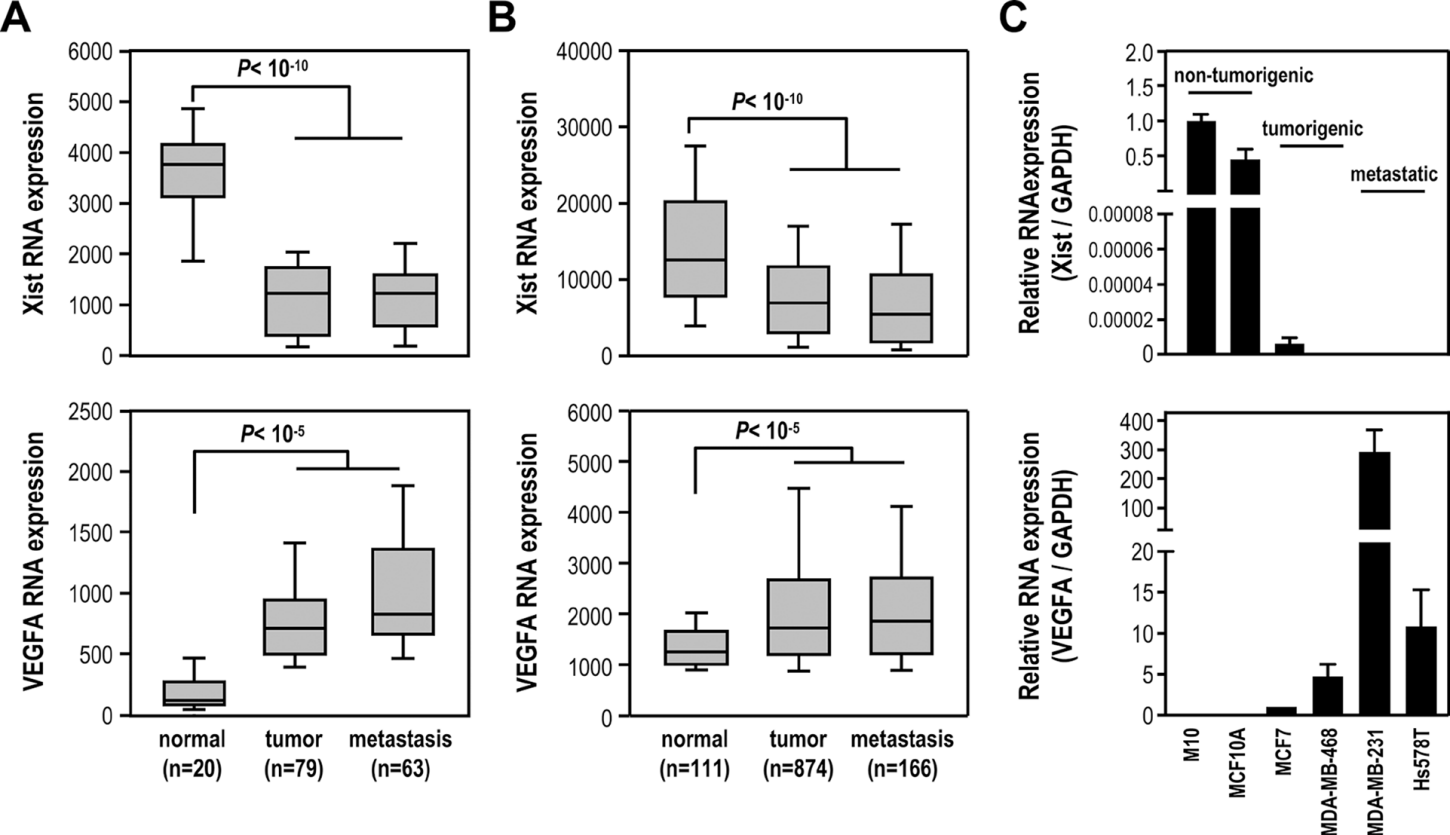
*Xist* expression is significantly reduced in breast cancer cell lines and breast cancer samples (**A**–**B**) Analysis of *Xist* and *VEGFA* expression levels in breast normal and tumor samples using the GEO and the TCGA data sets. (**C**) Quantification RT-PCR of *Xist* and *VEGFA* expression in non-tumorigenic breast cell lines and breast cancer cell lines (*n* = 3).

We investigated *Xist* expression in breast cancer using public data sets. Analysis of microarray data sets (GSE5764, GSE5460 and GSE14017) found *Xist* was significantly reduced in tumor and metastasis samples, compared to normal tissue, and inversely correlated with a positive control *VEGFA* expression profile (Figure 1A). Analysis of RNA-sequencing data sets from The Cancer Genome Atlas (TCGA) showed similar results (Figure 1B), suggesting *Xist* expression was downregulated in breast tumor and its expression in both tumor and metastasis samples was similar. We further examined *Xist* levels in non-tumorigenic (M10 and MCF10A), tumorigenic (MCF7 and MDA-MB-468), and metastatic (MDA-MB-231 and Hs578T) breast cell lines by quantitative RT-PCR analysis. Interestingly, *Xist* levels in MDA-MB-468, MDA-MB-231 and Hs578T cells were undetectable, while MCF7 cells expressed very low *Xist* levels, compared to M10 and MCF10A cells (Figure 1C). These results inversely correlated with a positive control VEGFA (Figure 1C). Our results suggested that *Xist* expression was decreased in breast tumor samples and breast cancer cell lines.

### *Xist* knockdown increases cell viability via AKT activation

**Figure 2:**
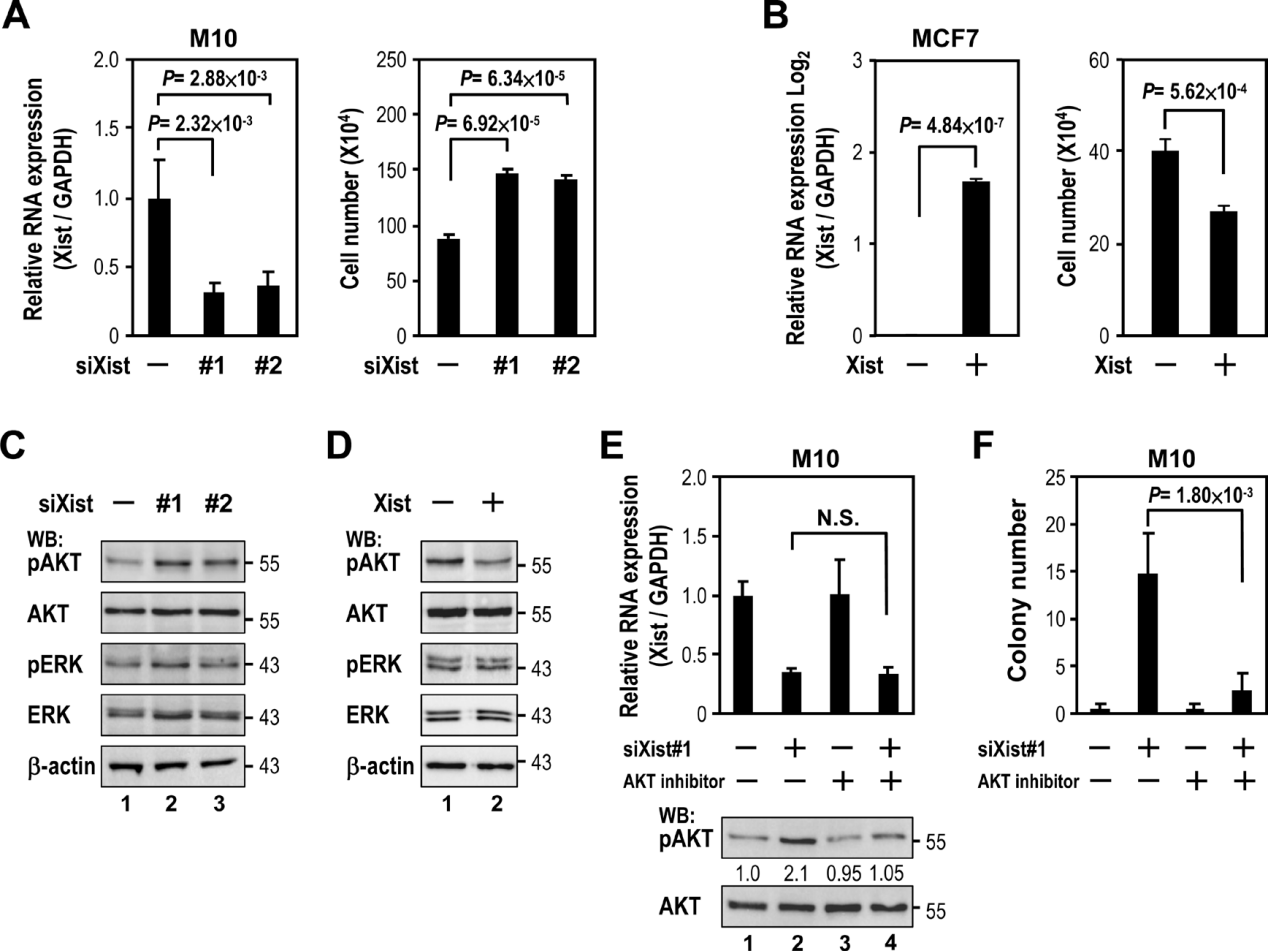
*Xist* downregulation increases cell viability via AKT activation (**A**) M10 cells were transfected with 10 nM specific siRNA oligonucleotides against *Xist* using RNAiMAX then subjected to quantitative RT-PCR (left panel) and viability assay (right panel). (**B**) MCF7 cells were transfected with *Xist* plasmid using Lipofectamine 3000 then subjected to quantitative RT-PCR (left panel) and viability assay (right panel). (**C–D**) Lysates from transfected M10 and MCF7 cells were immunoblotted with indicated antibodies. (**E**) Quantification RT-PCR and Western blotting of lysates from M10 cells transfected with *Xist* siRNA followed by AKT inhibitor treatment (250 nM, 2 days). Phospho- and non-phospho- AKT band intensity was quantified by densitometry. The ratio of phospho- to non-phospho protein is indicated after normalization to mock transfection and treatment samples. (**F**) Quantitative colony formation analysis of M10 cells transfected with *Xist* siRNAs and treated with or without 250 nM AKT inhibitor. Error bars: mean ± SD, *n* = 3.

Our findings of *Xist* expression in association with breast cancer led us to test the effect of *Xist* on cell viability, using knockdown and overexpression of *Xist* in M10 and MCF7 cells, respectively. Depletion of *Xist* in M10 cells resulted in increased cell viability (Figure 2A, right panel). In contrast, *Xist* overexpression in MCF7 cells reduced cell viability (Figure 2B, right panel). These results suggested downregulation of *Xist* expression promoted breast cancer cell viability.

We next explored the cellular pathway(s) of cell viability regulated by *Xist*. Since both RAS/ERK and PI3K/AKT pathways are the most frequently dysregulated signaling pathways associated with breast cancer cell growth and viability [16], we tested whether alteration of *Xist* levels modulated both pathways in breast cells. *Xist* knockdown by siXist increased phospho-AKT (pAKT) levels in M10 cells (Figure 2C). Conversely, *Xist* overexpression decreased phospho-AKT in MCF7 cells (Figure 2D). Notably, phospho-ERK was not altered by either *Xist* knockdown or overexpression (Figure 2C and 2D). These results suggested a role for the AKT pathway in cell viability regulated by *Xist*.

We reasoned that if AKT activation is involved in the upregulation of cell viability by *Xist* knockdown, inhibition of AKT activity should attenuate the effect of *Xist* knockdown on cell viability. Treatment of AKT inhibitor decreased *Xist* knockdown-elicited AKT phosphorylation in M10 cells (Figure 2E, lanes 2 and 4). Of note, AKT inhibitor treatment did not alter *Xist* expression levels (left panel, lanes 2 and 4). Under such treatment, cell viability of M10 cells upregulated by *Xist* knockdown was also attenuated (Figure 2F, lanes 2 and 4). These data suggested that AKT activation mediated *Xist* knockdown-elicited cell viability regulation.

### Negative correlation between *Xist* and phospho-AKT levels in clinical breast cancer

**Figure 3:**
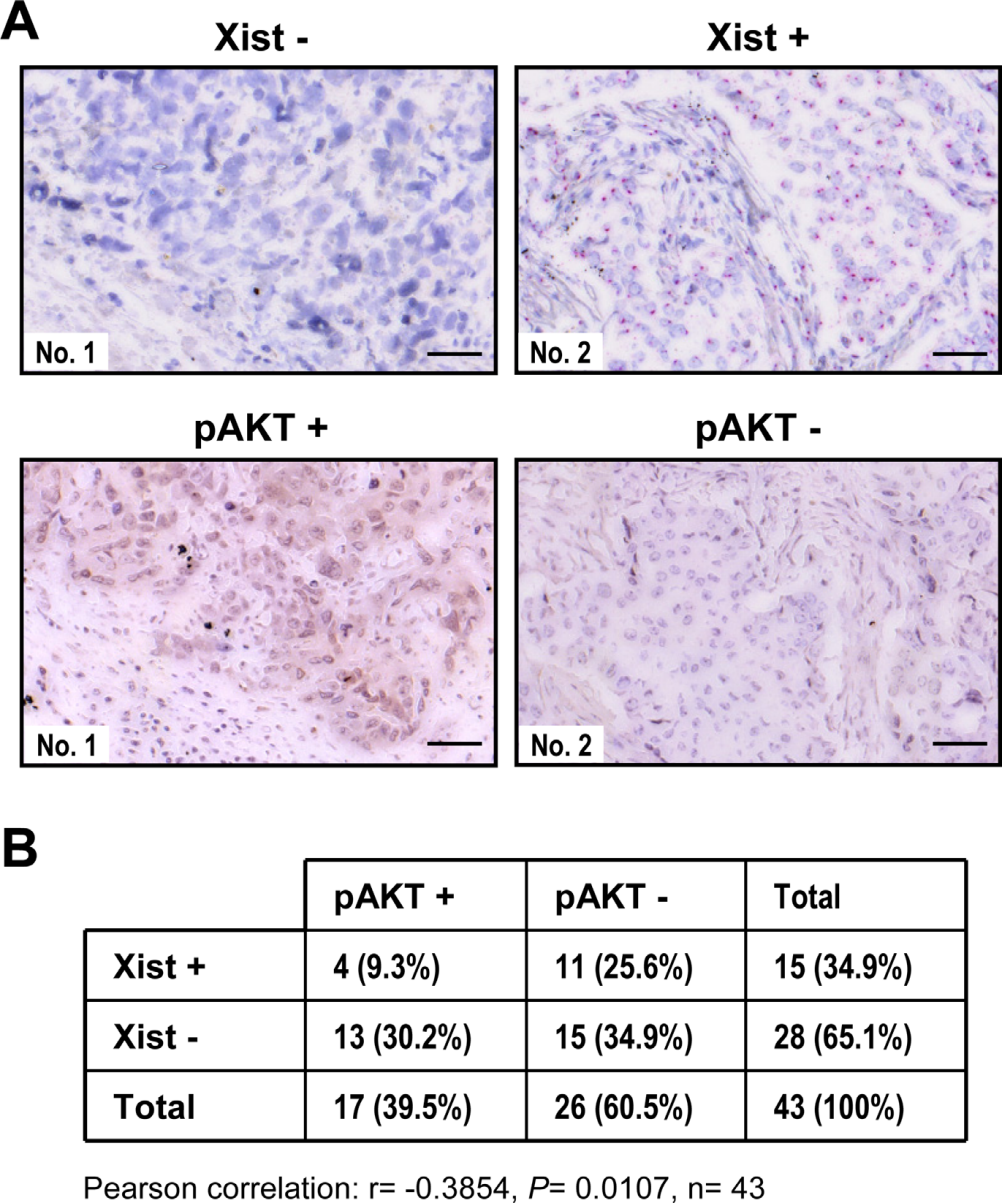
Inverse correlation between *Xist* and phospho-AKT in clinical breast samples (**A**) Immunohistochemistry to detect phospho-AKT (pAKT) (brown) and RNA *in situ* hybridization to visualize *Xist* (red) in serial sections of each breast cancer sample. Scale bar = 50 um. (**B**) Regression analysis of the correlation of *Xist* and pAKT levels.

Our finding that *Xist* negatively regulated pAKT levels in breast cell lines led us to examine whether an inverse correlation between *Xist* and pAKT occurred in clinical breast samples. As expected, immunohistochemistry (IHC) and RNA *in situ* hybridization analyses demonstrated that pAKT levels were higher in *Xist-*negative breast cancer tissue, while *Xist*-positive samples yielded lower pAKT levels (Figure 3A), suggesting an inverse correlation between *Xist* and pAKT levels. These data were supported by analysis of additional breast cancer samples (Figure 3B). These results, in conjunction with the above-mentioned cell line studies, suggested that decreased *Xist* levels resulted in an increase of pAKT levels in breast tumor.

### Association of Jpx in regulation of *Xist* expression in breast cancer

We next investigated whether the reduction of *Xist* expression in breast cancer could be derived from genomic deletion or epigenetic alteration. *Xist* genomic alteration in breast tumors was analyzed using GEO data sets (GSE26232). We did not observe copy number loss occurring in or near *Xist* locus, including *Jpx* and *Txis* loci (Supplementary Figure S1). These data excluded the possibility of *Xist* locus deletion in breast cancer.

**Figure 4:**
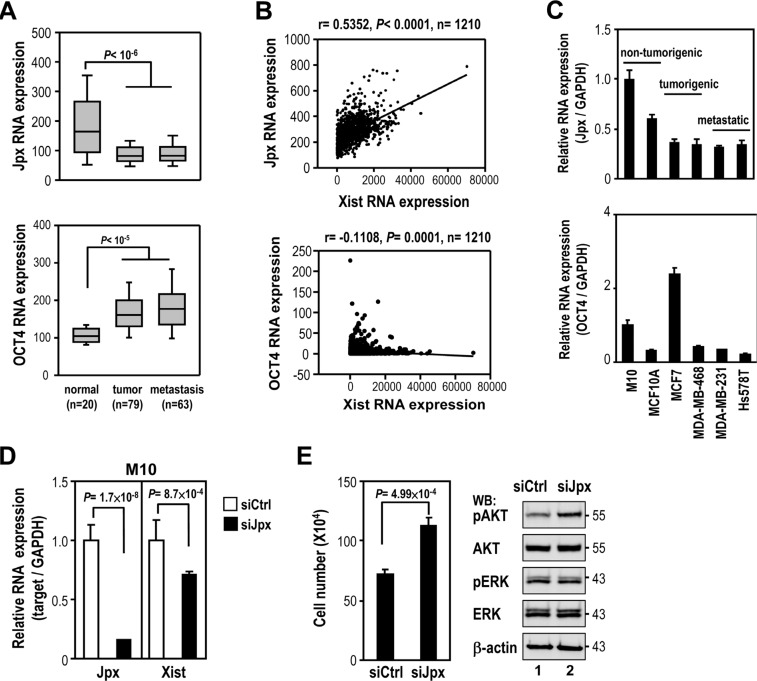
Association of *Jpx* in regulation of *Xist* expression in breast cancer (**A**) Coexpression analysis of *Jpx* and *OCT4* levels in breast normal and tumor samples using GEO data sets. (**B**) Coexpression analysis of *Xist* with *Jpx* or *OCT4* in clinical breast samples using TCGA data sets. (**C**) Quantitative RT-PCR of *Jpx* and *OCT4* expression in non-tumorigenic breast cell lines and breast cancer cell lines. (**D**–**E**) M10 cells were transfected with 10 nM specific siRNA oligonucleotides against *Jpx* using RNAiMAX then subjected to quantitative RT-PCR, viability assay and Western blot. Error bars: mean ± SD, *n* = 3.

Previous studies showed that *Xist* expression was positively controlled by *Jpx* lncRNA [17] and negatively regulated by *Tsix* lncRNA and transcription factors, such as PRDM14, OCT4, SOX2, REX1 and NANOG [10]. Bioinformatics analyses was used to test whether these lncRNAs and transcription factors regulated *Xist* in clinical breast samples. Analysis of GEO data sets revealed that *Jpx* expression was also reduced in breast tumor and metastatic tissue (Figure 4A, upper panel), suggesting possible *Jpx* regulation of *Xist* expression. *Tsix* expression was also decreased in breast tumor, excluding the possibility of *Tsix* in regulating *Xist* expression (Supplementary Figure S2A). Furthermore, the expression of OCT4, but not PRDM14, SOX2, REX1 or NANOG was higher in breast tumor and metastasis samples (Figure 4A, bottom panel, and Supplementary Figure S2B–S2E), suggesting negative regulation of *Xist* by OCT4.

We further analyzed whether *Xist* expression was associated with the expression of *Jpx* or OCT4 in breast cancer by TCGA data set. Interestingly, *Xist* and *Jpx* expression showed a robust positive correlation, while a negative correlation between *Xist* and OCT4 expression was relatively weak (Figure 4B). Accordingly, we found reduced *Jpx* expression in breast cancer cell lines, compared to non-tumorigenic cells (Figure 4C), correlating with *Xist* expression in these cell lines. Notably, OCT4 expression was elevated only in MCF7, but not in MDA-MB-468, MDA-MB-231 and Hs578T cell lines. These results implicated *Jpx* control of *Xist* expression in breast cancer.

Because *Jpx* positively modulates *Xist* expression [18], *Jpx* knockdown should reduce the negative effect of *Xist* on cell viability and AKT phosphorylation. As expected, treatment of *Jpx* siRNA reduced *Xist* expression in M10 cells (Figure 4D), correlating with increased levels of cell number and phospho-AKT (Figure 4E). These data suggest that reduced *Jpx* expression enhanced cell viability and AKT phosphorylation through downregulation of *Xist* in breast cancer.

### *Xist* knockdown-elicited AKT phosphorylation is via transrepression of PHLPP1 expression by HDAC3

**Figure 5:**
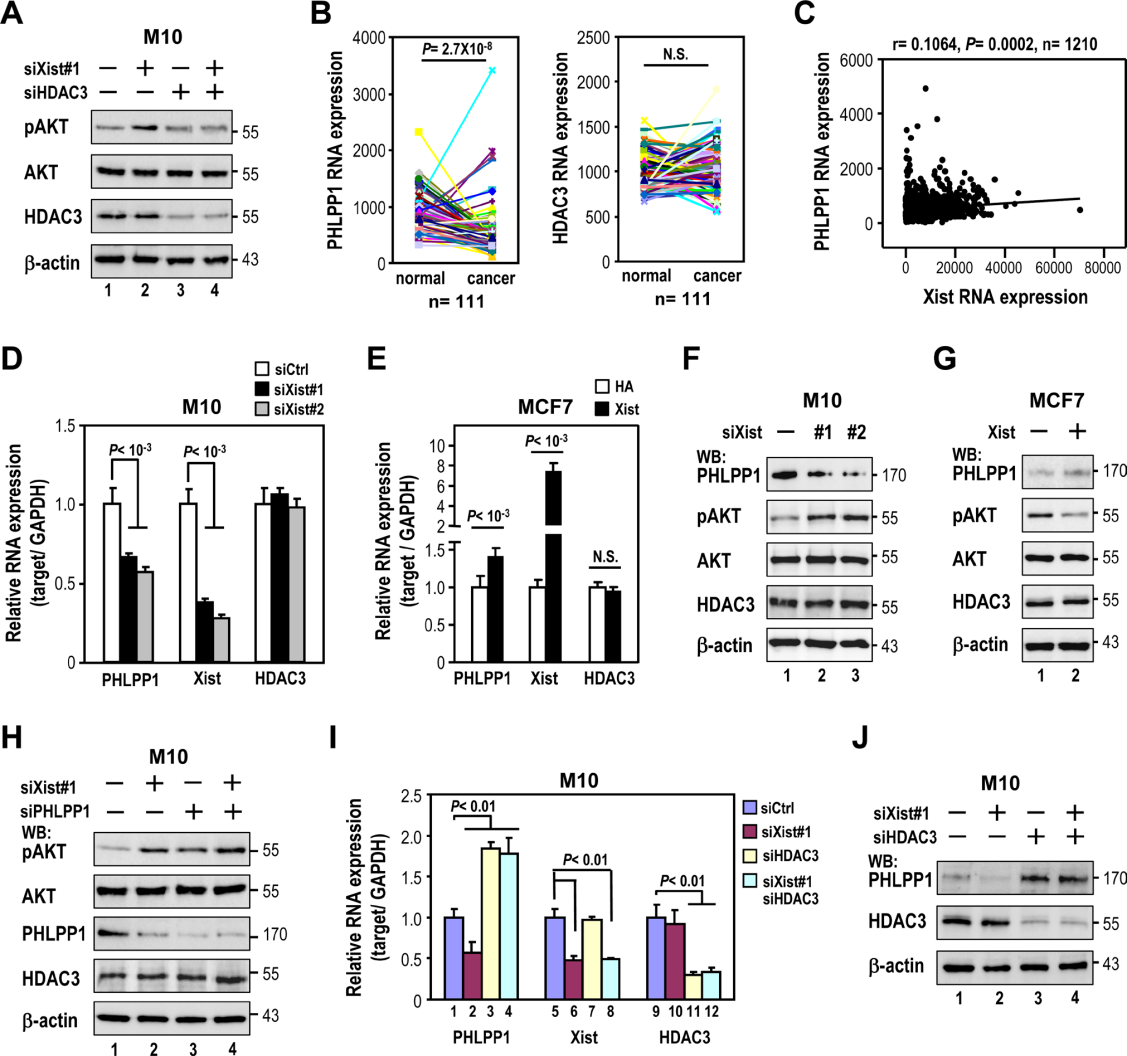
*Xist* knockdown-elicited AKT phosphorylation is via transrepression of PHLPP1 expression by HDAC3 (**A**) Western analysis of M10 cells transfected with *Xist* siRNA and/or HDAC3 siRNA with indicated antibodies. (**B**) Analysis of *PHLPP1* and *HDAC3* expression by RNA-seq from 111 pairs of matched normal breast and breast cancer tissues using TCGA data sets. (**C**) Coexpression analysis *Xist* and *PHLPP1* in clinical breast samples using TCGA data sets. (**D** and **F**) M10 cells were transfected with 10 nM specific siRNA oligonucleotides against *Xist* using RNAiMAX then subjected to quantitative RT-PCR analysis (D) and Western blotting (F). (**E** and **G**) MCF7 cells were transfected with *Xist* plasmid using Lipofectamine 3000 then subjected to quantitative RT-PCR analysis (E) and immunoblotting (G). (**H**–**J**). M10 cells were transfected with *Xist* siRNA and/or HDAC3 or PHLPP1 siRNA using RNAiMAX, then subjected to quantitative RT-PCR and Western analyses. Error bars: mean ± SD, *n* = 3.

Although *Xist* reduction is associated with increased pAKT in breast cancer, the molecular mechanism for such regulation is unknown. A recent report suggested *Xist* repressed gene expression on X-chromosome in female cells via recruitment of HDAC3 [12]. We questioned whether HDAC3 was involved in *Xist*-regulated AKT activation. Depletion of HDAC3 in M10 cells decreased the effect of *Xist* knockdown on pAKT induction (Figure 5A, lane 4 versus lane 2), suggesting HDAC3 mediated *Xist* knockdown-elicited AKT activation.

We further investigated the role of HDAC3 in the regulation of AKT activation. Recent research showed HDAC3 knockout increased the expression of the PH domain and leucine-rich repeat phosphatase 1 (PHLPP1) in chondrocytes to suppress AKT phosphorylation [19]. Interestingly, our TCGA data set analysis showed expression levels of PHLPP1, but not HDAC3, were decreased in breast cancer tissues compared to adjacent normal tissues (Figure 5B). Furthermore, PHLPP1 expression was positively associated with *Xist* expression (Figure 5C). These findings led us to test whether *Xist* expression could modulate PHLPP1 expression. Depletion of *Xist* in M10 cells decreased PHLPP1 expression levels (Figure 5D), while overexpression of *Xist* in MCF7 cells increased it (Figure 5E). Notably, neither knockdown nor overexpression of *Xist* altered HDAC3 expression (Figure 5D and 5E). We further confirmed the effect of *Xist* on PHLPP1 expression by Western analysis (Figure 5F and 5G). More importantly, the effect of the *Xist* knockdown on the increase in pAKT levels was significantly reduced in PHLPP1-depleted cells (Figure 5H, lanes 3 and 4 versus lanes 1 and 2). These results suggested *Xist*-elicited induction of phospho-AKT levels occurred via downregulation of PHLPP1 expression.

If HDAC3 is involved in the downregulation of PHLPP1 expression by *Xist* knockdown, depletion of HDAC3 levels should attenuate the negative effect of *Xist* knockdown on PHLPP1 expression. As expected, silencing of HDAC3 blocked the reduction of the PHLPP1 expression in *Xist-*depleted cells (Figure 5I and 5J, lanes 3 and 4 versus lanes 1 and 2) These results suggested that *Xist* knockdown-elicited downregulation of the PHLPP1 expression was HDAC3-dependent.

### Depletion of *Xist* or SPEN increases HDAC3 recruitment to PHLPP1 promoter

**Figure 6:**
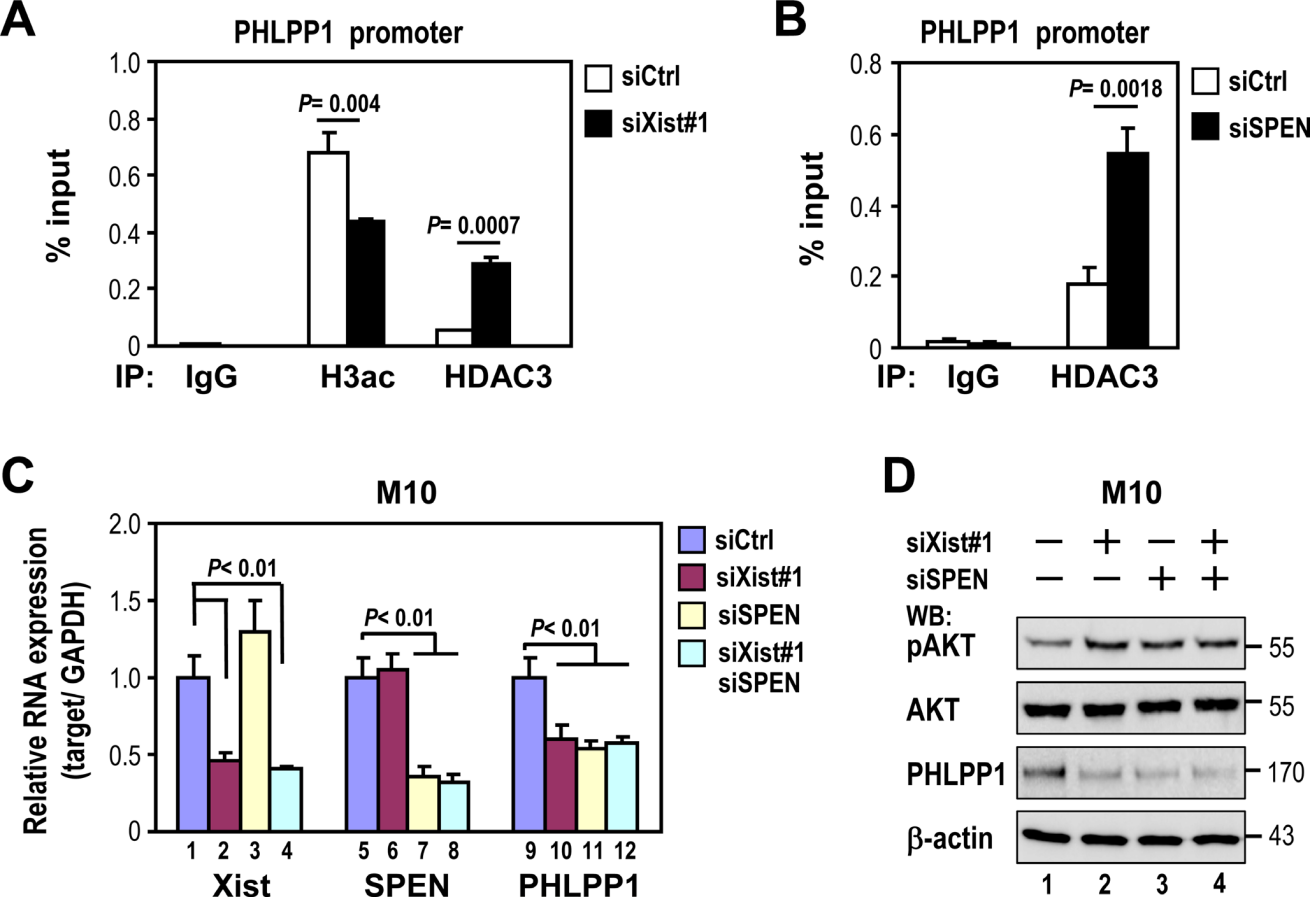
Depletion of *Xist* or SPEN increases HDAC3 recruitment to the PHLPP1 promoter and decreases PHLPP1 expression (**A** and **B**) M10 cells were transfected with 10 nM specific siRNA oligonucleotides against *Xist* or SPEN using RNAiMAX then subjected to chromatin immunoprecipitation (ChIP). ChIP analyses were performed with antibodies against HDAC3, acetylated histone H3 (H3-ac) or an IgG control. Subsequent qPCR analysis was carried out by primers specific for PHLPP1 promoter. Input represents 1% of the chromatin used for immunoprecipitation. (**C** and **D**) M10 cells were transfected with *Xist* siRNA and/or SPEN siRNA using RNAiMAX then subjected to quantitative RT-PCR (C) and Western analyses (D). Error bars: mean ± SD, *n* = 3.

Because HDAC3 levels were not altered by *Xist* expression, we hypothesized that HDAC3 recruitment to repress PHLPP1 promoter activity would be altered by *Xist*. We examined the effect of *Xist* on HDAC3 recruitment to the PHLPP1 promoter by chromatin immunoprecipitation (ChIP). Notably, HDAC3 recruitment to the PHLPP1 promoter region was increased in *Xist*-depleted M10 cells (Figure 6A), and correlated with a decreased level of histone 3 acetylation (H3ac) in this promoter region. These results suggested that *Xist* negatively regulated HDAC3 targeting to the PHLPP1 promoter.

Recent research showed that *Xist* indirectly recruited HDAC3 via SPEN for XCI establishment [12]. This finding led us investigate whether *Xist* sequesters HDAC3 from the PHLPP1 promoter via SPEN. CHIP analysis was used to determine if SPEN knockdown also altered HDAC3 recruitment to the PHLPP1 promoter. Similar to *Xist*, SPEN knockdown increased the level of HDAC3 recruitment to the PHLPP1 promoter region in M10 cells (Figure 6B). These results suggested that both *Xist* and SPEN retained HDAC3 in X-chromosome, thereby reducing HDAC3 recruitment to the PHLPP1 promoter region.

If SPEN, like *Xist*, is involved in sequestering HDAC3 from the PHLPP1 promoter region, depletion of SPEN levels should attenuate the expression of PHLPP1. As expected, SPEN knockdown decreased PHLPP1 expression (Figure 6C, lane 11 and Figure 6D, 3_rd_ panel, lane 3), which also correlated with an increase of pAKT (Figure 6D, top panel, lane 3). Notably, SPEN knockdown blocked the effect of *Xist* knockdown on both PHLPP1 downregulation and pAKT upregulation (Figure 6C, lanes 11 and 12 versus lanes 9 and 10; Figure 6D, lanes 3 and 4 versus lanes 1 and 2). These results provided additional support that *Xist* positively regulates PHLPP1 expression via sequestering HDAC3 from the PHLPP1 promoter.

## DISCUSSION

The molecular basis of *Xist* as a tumor suppressor lncRNA is largely unclear. In the present study, we demonstrate an inverse relationship of *Xist* and phospho-AKT levels in breast cancer tissues. More importantly, *Xist* knockdown-mediated upregulation of AKT phosphorylation occurred via suppression of PHLPP1 expression by HDAC3. Our findings suggest that *Xist* functions as a tumor suppressor through inhibition of AKT activation in breast cancer. A recent study reported that conditional deletion of *Xist* from HSCs in female mice lead to the development of highly aggressive MDS/MPN [5]. AKT was also prominently activated in MDS/MPN [20]. Our findings of *Xist*-enhanced PHLPP1 expression in decreasing AKT phosphorylation may also provide a possible molecular basis for *Xist* loss-associated MDS/MPN. Indeed, PHLPP1 expression was decreased in the microarray analysis of *Xist*-deficient HSCs in mice [5]. Furthermore, analysis of GEO data set also revealed decreased levels of PHLPP1 in MDS patients, correlating with reduced *Xist* levels (data not shown). Our results that *Xist* negatively modulated AKT activation via PHLPP1 regulation suggest a tumor suppressor role of *Xist* in breast cancer and MDS/MPN.

The loss of *Xist* expression could induce the reactivation of X-linked genes, which may contribute to breast cancer progression. Recent studies identified X-linked genes in X chromosome reactivation, including HDAC8 and TBL1X [7]. HDAC8 was suggested to activate breast cancer stem cell-like properties and increase cell invasion [21, 22]. Although X-linked gene reactivation might contribute to breast cancer progression [23], our finding of PHLPP1 expression regulated by *Xist* levels in downregulating AKT phosphorylation provides evidence that non-X-linked gene expression regulated by *Xist* may also play an important role in breast cancer progression. Currently, the mechanism of *Xist* regulation of PHLPP1 remains largely unclear. While *Xist* could indirectly recruit HDAC3 via SPEN/SHARP and SMRT proteins for XCI establishment [12, 13], our findings that knockdown of either *Xist* or SPEN increased HDAC3 recruitment to the PHLPP1 promoter region for gene suppression led us to hypothesize that *Xist* reduction may release HDAC3 protein from inactive X-chromosome, thereby increasing the HDAC3 pool for PHLPP1 promoter targeting. In this scenario, *Xist* could function as an lncRNA decoy to titrate HDAC3 from the PHLPP1 promoter. A similar scenario of lncRNA decoys, such as *PANDA*, has been well documented [24]. PANDA was shown to inhibit apoptotic gene expression through sequestering transcription factor NF-YA, preventing NF-YA's transactivation of apoptotic program upon DNA damage [25].

While we demonstrated that *Xist* expression in breast cancer was associated with *Jpx* expression, very little is known about *Jpx* expression downregulation in breast cancer. Two possible scenarios could be considered: A recent report showed that *Jpx* and *Xist* promoters were fully hypermethylated in breast cancer cell lines [26]. Thus, one possible scenario is that reduction of both *Jpx* and *Xist* expression in breast cancer may resulted from hypermethylation. Research also showed that chromosome conformation at the *Tsix* topologically associating domain could regulate *Tsix* transcriptional status [27]. In this scenario, decreased *Jpx* expression in breast cancer may be due to altered chromosome conformation. These two possibilities are not mutually exclusive. Further studies are required to elucidate *Jpx* downregulation in breast cancer.

In summary, our data suggest a tumor suppressor role of *Xist* in inhibiting AKT activation via regulation of non-X-chromosome gene PHLPP1 expression. In addition to the molecular basis of *Xist* in modulating AKT activation, these findings of decreased *Xist* levels and increased AKT phosphorylation may potentially be considered as markers for breast cancer diagnosis and therapeutic intervention.

## MATERIALS AND METHODS

### Analysis of public data sets

Affymetrix HG U133 plus 2.0 arrays data sets (GSE5764, GSE5460 and GSE14017) and Affymetrix Genome-Wide Human SNP 6.0 array data sets (GSE26232) were downloaded from NCBI GEO [28–30]. Expression data derived from downloaded CEL files were normalized using the invariant set and converted into intensity using dChip (www.dchip.org) software [31]. Processed RNA-sequencing data of TCGA breast invasive carcinoma (BRCA) dataset were obtained from the TCGA Data Portal (https://tcga-data.nci.nih.gov/tcga/) [32].

### Quantitative RT-PCR (RT-qPCR)

Total RNA was extracted as previously described [33]. Total RNA (5 μg/sample) was reverse-transcribed using Maxima H Minus First Strand cDNA Synthesis Kit (Thermo Scientific, Vilnius, Lithuania). Quantitative real-time PCR was performed using Luminaris Color HiGreen Low ROX qPCR Master Mix (Thermo Scientific, Vilnius, Lithuania) and an ABI 7500 sequence detection system (Life Technologies, California, USA). Purified RNA was subjected to RT-qPCR, using specific primers: *Xist* (NR_001564): 5′- GCATAACTCGGCTTAGGGCT-3′ and 5′-TCCTCTGCCTGACCTGCTAT-3′, *VEGFA* (NM_001 025366): 5′- CGCAAGAAATCCCGGTATAA -3′and 5′- TCT CCGCTCTGAGCAAGG-3′, *GAPDH* (NM_002046): 5′- TCTTTTGCGTC GCCAGCCGAG -3′and 5′- TGA CCAGGCGCCCAATACGAC -3′, *Jpx* (NR_024582): 5′-AG ACTTAAGATGGCGGCGTT-3′and 5′-TGGACTCAT AC TTCGGACGC-3′, and *Tsix* (NR_003255): 5′-GTGTAGG TGGTTCCCC AAGG-3′ and 5′-AATGACTGACCACTG CTGGG-3′, OCT4 (NM_002701): 5′- TGCAGCAGATCAG CCACAT-3′ and 5′-CTCGGACCACATCCTTCTCG-3′, *HDAC3* (NM_003883): 5′- GGCCTATTTCTACGACCCCG -3′ and 5′-TGGTATGGCTTGAAGACGATCA-3′, *PHLPP1* (NM_194449): 5′-ACTCCAACTGCATCGAGGTC-3′ and 5′-AGCTCAG GTCCACACACTTG-3′, SPEN (NM_015 001): 5′-GGAGACTTGATGGGGCTTCA-3′ and 5′-GAT CAA ATCCTCCCGTCCCC-3′.

### Cell culture, plasmids, RNA interference and transfection

All breast cancer cell lines were cultured in specific medium with 10% fetal bovine serum (FBS). MCF-7, MDA-MB-468, Hs578T cells were cultured in DMEM; MDA-MB-231 and M10 cells in RPMI and MEM medium, respectively, and MCF10A cells in DMEM/F12 with 10 μg/ml insulin, 20 ng/ml EGF, 100 ng/ml choleratoxin, 0.5 μg/ml hydrocortisone and 5% horse serum. *Xist* plasmid pCMV-Xist-PA was a gift from Rudolf Jaenisch (Addgene plasmid # 26760) [34]. Specific siRNA oligonucleotides against *Xist*, *Jpx*, HDAC3 and negative control were synthesized by Life Technologies. The siRNAs sequences were: siXist#1: GUAUCCUAUUUGCACGCUAtt, siXist#2: GCCCUU CUCUUCGAACUGUtt, siJpx: GAAAAGGAAUAAA AUCAAAtt, siHDAC3: CCAAGAGUCUUAAUGCCU Utt, and siSPEN: CACGCAGAGUUACCGAAAAtt. Transfections were performed using RNAiMAX or Lipofectamine 3000 (Life Technologies).

### Cell viability analysis and colony formation assay

Viability of cells transfected with indicated siRNA or plasmid constructs was determined by trypan blue dye exclusion. Colony formation assay was performed with M10 cells transfected with siXist#1 and pTK-hyg, and treated with or without 250 nM AKT inhibitor (A6730, Sigma, St. Louis, USA), further selected with hygromycin (Life Technologies) for 10 days followed by crystal violet staining.

### Protein extraction and Western blot analysis

Total protein was extracted as previously described [35]. Briefly, samples were loaded onto SDS-PAGE gels (8∼10%, 40 μg/lane), transferred onto Protran Transfer Membrane (NBA085C001EA, PerkinElmer, Boston, USA), probed with antibodies against: β-actin (A5441, Sigma), PHLPP1 (ab71972, Abcam, Cambridge, UK), HDAC3 (17–1 0238, Millipore, Temecula, USA), acetyl-histone H3 (06–5 99, Millipore), phosphorylated-ERK (9101, Cell Signaling Technology), ERK (4695, Cell Signaling Technology), phosphorylated-AKT (4060, Cell Signaling Technology) and AKT (4691, Cell Signaling Technology), and analyzed with a Las-4000 imaging system (Fujifilm, Valhalla, USA).

### Immunohistochemistry (IHC) analysis and RNA *in-situ* hybridization (ISH)

Tissue array slides were purchased from SUPER BIO CHIPS, Seoul, Korea with Institute Review Board approval. Sections were deparaffinized and rehydrated with xylene and a series grade of alcohol. Epitope retrieval was carried out in a 10 mM citrate buffer (pH 6.0, 15 min, 99°C) using a hot plate, followed by inactivation of endogenous peroxidase with H_2_O_2_ and incubation with phosphorylated-AKT (4060, Cell Signaling Technology, 1 h, room temperature). Immunostaining was performed according to standard procedures. Slides were stained with Vectastain Elite ABC kit (Vector Lab, Burlingame, CA), developed using DAB (brown precipitate, Vector Lab), and visualized by light microscopy (Olympus, Allentown, USA). ISH was performed as previously described [36]. Sections were hybridized with custom-designed QuantiGene ViewRNA probes against *Xist*. Bound probes were amplified using PreAmp and Amp molecules (all reagents from Affymetrix, Santa Clara, USA). Multiple Label Probe oligonucleotides conjugated to alkaline phosphatase were added and Fast Red Substrate was used to produce signal (Cy3). Images of histological samples were digitalized using Mirax Scan. Samples were grouped into four categories based on staining intensity: none (0), weak (+1), medium (+2) and strong (+3); all samples negative (stain intensity 0 and +1) and positive (stain intensity +2 and +3) were included in the analysis.

### Chromatin immunoprecipitation quantitative PCR (ChIP-qPCR) assay

ChIP was performed as previously described [33]. ChIP product was analyzed by quantitative real-time PCR using the Applied Biosystem 7500 Real-Time PCR System. Experiments were done in triplicate. A fraction (1%) of the sonicated chromatin was set aside as input control before antibody affinity manipulations. Percent input was calculated by 100 × 2^(Ct^adjusted Input^ - Ct^IP^). Purified DNA was subjected to qPCR, using specific primers for mouse *PHLPP1* promoter: 5′-AAGCTGGAGTGCGGGTAAAA-3′ and 5′- CCTCTGCCGTTGAACTCTGT-3′.

### Statistical analysis

Analyses were performed with SigmaPlot software. Significance (*p* ≤ 0.05) was ascertained using a Student's *t-*Test.

## SUPPLEMENTARY MATERIALS


