# Supplementary material for: Xist reduction in breast cancer upregulates AKT phosphorylation via HDAC3-mediated repression of PHLPP1 expression
Source: Oncotarget. 2016 May 27;7(28):43256–66. doi: 10.18632/oncotarget.9673 (PMC5190021; doi:10.18632/oncotarget.9673)
Supplement: Supplementary file 1 [file oncotarget-07-43256-s001.pdf]

Xist reduction in breast cancer upregulates AKT phosphorylation via HDAC3-mediated repression of PHLPP1 expression

Supplementary Materials

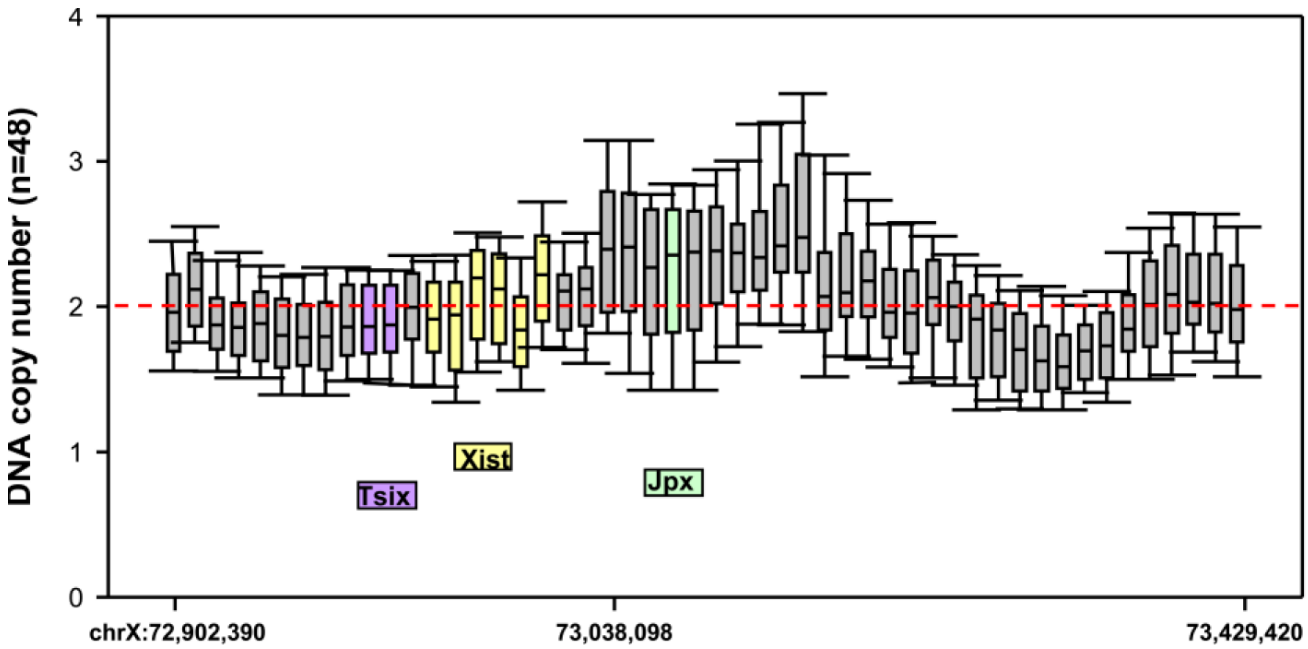

Supplementary Figure S1: DNA copy number at the locus of *Xist*, *Tsix* and *Jpx* in clinical breast cancers. The regions displayed include chromosome X from 72,902,390 to 73,429,420. The locations of *Xist*, *Tsix*, and *Jpx* are indicated.

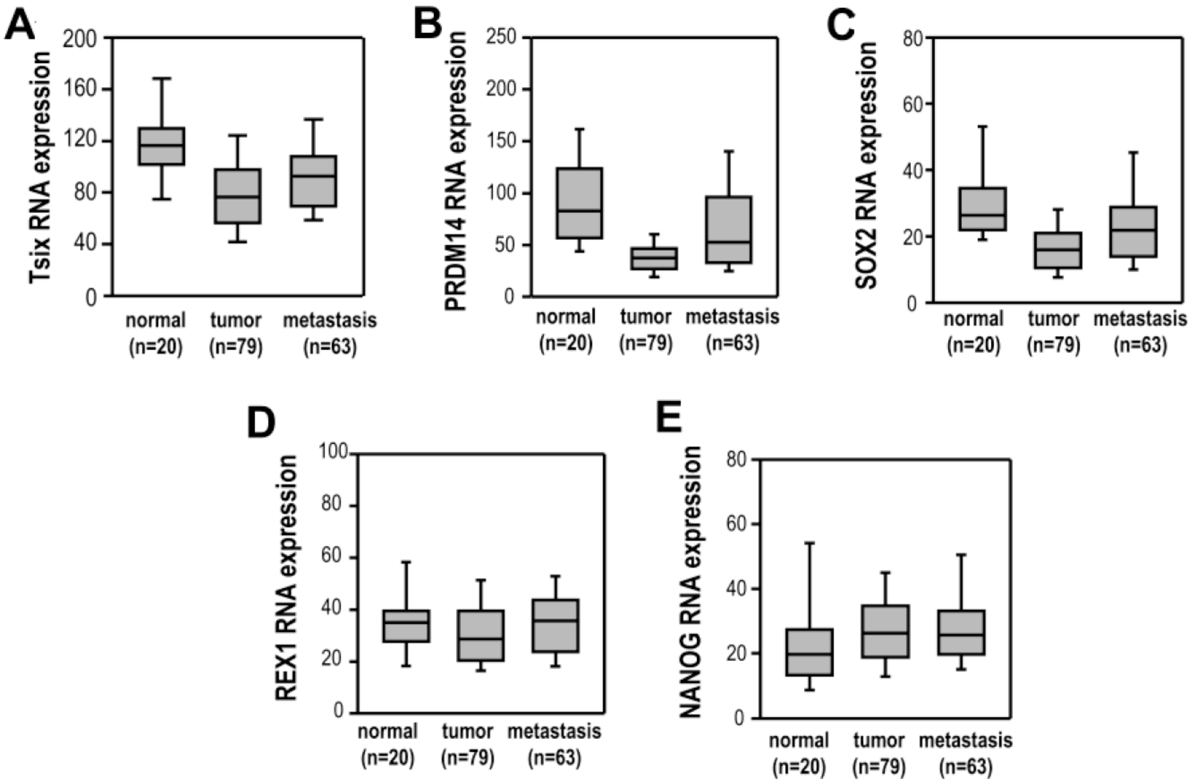

Supplementary Figure S2: The expression levels of the *Xist* negative regulators were not increased in breast cancer samples. **(A–E)** Analyses of *Tsix, PRDM14, SOX2, REX1 and NANOG* expression levels in breast normal and tumor samples using the GEO data sets.
